# Inflammatory cytokines and organ dysfunction associate with the aberrant DNA methylome of monocytes in sepsis

**DOI:** 10.1186/s13073-019-0674-2

**Published:** 2019-10-29

**Authors:** Clara Lorente-Sorolla, Antonio Garcia-Gomez, Francesc Català-Moll, Víctor Toledano, Laura Ciudad, José Avendaño-Ortiz, Charbel Maroun-Eid, Alejandro Martín-Quirós, Mónica Martínez-Gallo, Adolfo Ruiz-Sanmartín, Álvaro García del Campo, Ricard Ferrer-Roca, Juan Carlos Ruiz-Rodriguez, Damiana Álvarez-Errico, Eduardo López-Collazo, Esteban Ballestar

**Affiliations:** 1 Epigenetics and Immune Disease Group, Josep Carreras Research Institute (IJC), 08916 Barcelona, Spain; 2 Chromatin and Disease Group, Cancer Epigenetics and Biology Programme (PEBC), Bellvitge Biomedical Research Institute (IDIBELL), L’Hospitalet de Llobregat, 08908 Barcelona, Spain; 3 Innate Immunity Group, IdiPAZ, La Paz University Hospital, 28046 Madrid, Spain; 4 Emergency Department, IdiPAZ, La Paz University Hospital, 28046 Madrid, Spain; 5 Immunology Division, Vall d’Hebron University Hospital and Diagnostic Immunology Research Group Vall d’Hebron Research Institute (VHIR), 08035 Barcelona, Spain; 6 Intensive Care Department, Vall d’Hebron University Hospital, Shock, Organ Dysfunction and Resuscitation (SODIR) Research Group, Vall d’ Hebron Research Institute (VHIR), Universitat Autònoma de Barcelona, 08035 Barcelona, Spain; 7 Cardiac Post-Surgery Unit (UPCC), Vall d’Hebron University Hospital, 08035 Barcelona, Spain; 8 Tumor Immunology Lab, IdiPAZ, La Paz University Hospital, 28046 Madrid, Spain; 9 Center for Biomedical Research Network, CIBEres, Madrid, Spain

**Keywords:** Sepsis, DNA methylation, Cytokines, Endotoxin tolerance, Monocytes

## Abstract

**Background:**

Sepsis, a life-threatening organ dysfunction caused by a dysregulated systemic immune response to infection, associates with reduced responsiveness to subsequent infections. How such tolerance is acquired is not well understood but is known to involve epigenetic and transcriptional dysregulation.

**Methods:**

Bead arrays were used to compare global DNA methylation changes in patients with sepsis, non-infectious systemic inflammatory response syndrome, and healthy controls. Bioinformatic analyses were performed to dissect functional reprogramming and signaling pathways related to the acquisition of these specific DNA methylation alterations. Finally, in vitro experiments using human monocytes were performed to test the induction of similar DNA methylation reprogramming.

**Results:**

Here, we focused on DNA methylation changes associated with sepsis, given their potential role in stabilizing altered phenotypes. Tolerized monocytes from patients with sepsis display changes in their DNA methylomes with respect to those from healthy controls, affecting critical monocyte-related genes. DNA methylation profiles correlate with IL-10 and IL-6 levels, significantly increased in monocytes in sepsis, as well as with the Sequential Organ Failure Assessment score; the observed changes associate with TFs and pathways downstream to toll-like receptors and inflammatory cytokines. In fact, in vitro stimulation of toll-like receptors in monocytes results in similar gains and losses of methylation together with the acquisition of tolerance.

**Conclusion:**

We have identified a DNA methylation signature associated with sepsis that is downstream to the response of monocytes to inflammatory signals associated with the acquisition of a tolerized phenotype and organic dysfunction.

## Background

Sepsis is a life-threatening organ dysfunction caused by a dysregulated host response to infection [1]. Sepsis can induce acute kidney injury and multiple organ failures and represents the most common cause of death in intensive care units [2, 3]. The immune response during sepsis is complex and varies over time, with the concomitant occurrence of both pro-inflammatory and anti-inflammatory mechanisms [3]. Despite intense study, the cellular and molecular basis of human sepsis remains still unclear and effective therapies are lacking.

In many cases, sepsis survivors continue to succumb to secondary challenges, latent infections, or malignancies several years after the initial septic episode [4]. It has been recognized that sepsis leads to the acquisition of tolerance, a state of reduced responsiveness to subsequent stimulation after a primary bacterial insult that results in reduced cytokine production by monocytes and macrophages [5]. As a result, most patients with sepsis rapidly display signs of profound immunosuppression, associated with an increase in hypoxia inducible factor-1α expression that drives functional reprogramming [6]. Such immune reprogramming is in part due to disruption of homeostasis and defective cellular energy metabolism which underlies the inability to respond to secondary or further stimulation [7]. A number of mechanisms are involved in the homeostasis of the immune system, where monocytes are pivotal. These cells recognize pathogen patterns or intercept and phagocytose antigens, critical steps in eliminating bacterial infections, halting the increase in viral load, and eradicating certain neoplastic growths. Several monocyte subtypes are also crucial to the de-escalation of inflammation and in wound healing [8]. In vitro experiments mimicking septic conditions have shown that upon endotoxin re-challenge with Gram-negative bacteria or only lipopolysaccharide (LPS), the major component of the outer membrane of Gram-negative bacteria, tolerized monocytes/macrophages show a drastic downregulation of inflammatory cytokines (e.g., tumor necrosis factor [TNF] α, interleukin [IL]-6, IL-1β, IL-12) in parallel with the upregulation of anti-inflammatory cytokines like IL-10, transforming growth factor (TGF) β, and IL-1RA as compared to non-tolerized cells challenged with the same stimuli. These tolerant monocytes/macrophages also show an impaired antigen presenting capacity correlated with decreased expression of human leukocyte antigen (HLA)-DR and some costimulatory molecules [9] and upregulation of the immune checkpoint ligand PD-L1 [10].

The acquisition of endotoxin tolerance is accompanied by a remodeling of the epigenomic profiles [11–13]. Most studies have focused on histone modification changes. Following LPS stimulation of macrophages, toll-like receptor (TLR)-induced genes are categorized into two classes: tolerized and non-tolerized genes. Tolerized genes, which include inflammatory genes, show repressed expression whereas non-tolerized genes increase their levels. Transcriptional activation of non-tolerized genes is associated with high levels of histone acetylation and H3K4me3 at their promoters [11]. In addition, monocytes exposed to LPS showed changes in H3K27ac, H3K4me1, and H3K4me3 [12]. It has also been shown that during endotoxin tolerance, leukocytes display increased levels of repressive H3K9me2 mark at the promoter regions of the *IL1B* and *TNF* genes [14, 15]. Specifically, the H3K9 histone methyltransferase, G9a, is essential for silencing the *TNFA* gene [16].

In this context, DNA methylation changes have received less attention than histone modifications for several reasons. It has been mainly because DNA methylation has a more limited range of effects than histone modifications [17]. Nevertheless, DNA methylation changes are generally highly relevant for the biology of myeloid cells [18]. On the one hand, various studies have demonstrated the relevance of DNA (cytosine-5)-methyltransferase 3A (DNMT3A) and ten-eleven translocation methylcytosine dioxygenase 2 (TET2), both enzymes respectively essential for the de novo incorporation and oxidation/removal of methyl groups to cytosines, to the function of monocytes, dendritic cells, and macrophages [19, 20]. On the other hand, DNA methylation is generally associated with the stabilization of a transcriptional and functional state; thus, it is appealing to hypothesize that sepsis results in prolonged acquisition of DNA methylation changes of the monocytes well beyond the acute phase of sepsis, and perhaps contributing to stabilize the state of tolerance of monocytes. Most importantly, TET2 has a role in resolution of inflammation by recruiting HDAC2 to repress inflammatory genes [21] and to promote sepsis-induced emergency myelopoiesis [22].

In this study, we investigated DNA methylation changes in monocytes from individuals who have experienced an episode of sepsis. We performed DNA methylation profiling where we compared sorted monocytes from patients with sepsis and healthy controls. The analysis revealed the existence of significant DNA methylation differences between the two groups in CpG sites mapping at genes relevant for monocyte-related immune responses. Most importantly, we identified a significant relationship between DNA methylation data and IL-10 and IL-6 cytokine levels, which are significantly increased in patients with sepsis, as well as with organ dysfunction. We have determined that changes in DNA methylation are determined by TLR stimulation and the altered levels of inflammatory cytokines. Our findings also highlight the implication of TLR stimulation and cytokines under sepsis in establishing and perpetuating the dysregulated epigenetic signature and phenotype of monocytes.

## Methods

### Human samples

We selected and diagnosed patients with sepsis based on the Third International Consensus Definitions for Sepsis and Septic Shock (Sepsis-3) [1]. For each patient, we calculated the Sequential [Sepsis-related] Organ Failure Assessment (SOFA) score. The study included 14 patients with bacterial infections with SOFA ranging from 2 to 8. Patients were obtained from La Paz University Hospital and Vall d’Hebron University Hospital. Blood samples were collected at the first 12 h of sepsis diagnosis, which was confirmed using clinical and analytical data. The clinical data of the patients included in the study are summarized in Table 1 and Additional file 1: Table S1. We also studied a group of individuals with non-infectious systemic inflammatory response syndrome (SIRS), formed by 4 patients in the immediate postoperative period of cardiac surgery. In this group, the blood samples were obtained in the first 24 h of the postoperative period. Finally, we also included blood samples from 11 healthy controls collected from the blood donor service of La Paz University Hospital and Vall d’Hebron University Hospital. The Committee for Human Subjects of La Paz University Hospital (PIE2392) and Vall d’Hebron University Hospital (PR (ATR)122/2019) approved the study, which was conducted in accordance with the ethical guidelines of the 1975 Declaration of Helsinki. All samples were in compliance with the guidelines approved by the local ethics committee, and all patients (sepsis, SIRS, and healthy controls) received oral and written information about the possibility that their blood would be used for research purposes and signed informed consent.

**Table 1:** Summary of the patient cohorts in the study

	Healthy controls	SIRS-cardio patients	Septic patients	*p* value (sepsis vs control)	*p* value (sepsis vs SIRS)	*p* value (SIRS vs control)
*N*	11	4	14			
Age (mean ± SD)	51 ± 11.8	67.8 ± 5.8	74.6 ± 14.5	0.0010***	0.3662	0.0130**
Sex (% female)	27.3	25	57.1	0.1353	0.2568	0.9299
SOFA	0	3.8 ± 2.2	3.9 ± 2.0	N/A	0.8226	N/A
Basal cytokine levels						
TNFα (pg/ml) (mean ± SD)^†^	9.0 ± 17.0	2.7 ± 3.1	561.4 ± 2082.8	0.9774	0.7845	0.7396
IL6 (pg/ml) (mean ± SD)^†^	11.9 ± 13.1	123.8 ± 101.4	4273.4 ± 10,789.5	0.0002***	0.7906	0.0050***
IL10 (pg/ml) (mean ± SD)^†^	5.8 ± 5.1	81.6 ± 41.4	55.6 ± 72.9	0.0005***	0.1236	0.0050***

*p* values of continuous data were calculated using Mann-Whitney *U* test and categorical data were calculated using chi-squared test. Statistically significant tests are represented as **p < 0.01 and ***p < 0.005

^†^These correspond to the basal levels of the cytokines measured in the serum

### Purification of human monocytes from patients with sepsis, SIRS, and healthy control samples

Peripheral blood mononuclear cells (PBMCs) were obtained from blood by density gradient centrifugation using lymphocyte isolation solution (Rafer, Zaragoza, Spain). PBMCs were stained with CD14-PE (Miltenyi Biotec, Bergisch Gladbach, Germany), CD66b-APC (STEMCELL™ Technologies, Vancouver, Canada), and Fixable Viability Dye eFluor™ 520 (ThermoFisher Scientific, MA, USA), and then, cells were fixed with 2% formaldehyde methanol-free (ThermoFisher Scientific). Pure monocytes were isolated as CD14+CD66b− cells using flow cytometry sorting (MoFlo Astrios EQ, Beckman Coulter Spain, L’Hospitalet de Llobregat, Barcelona, Spain). Purified samples were pelleted and stored at − 80 °C.

For in vitro experiments, we obtained buffy coats from anonymous donors through the Catalan Blood and Tissue Bank (CBTB). The CBTB follows the principles of the World Medical Association (WMA) Declaration of Helsinki. Before providing blood sample, all donors received detailed oral and written information and signed a consent form at the CBTB. PBMCs were isolated by density gradient centrifugation. Then, PBMCs were resuspended in Roswell Park Memorial Institute (RPMI) Medium 1640 + GlutaMAXTM-1 (Gibco, Life Technologies, CA, USA) containing 10% human pooled serum (One Lambda, ThermoFisher Scientific Brand, Canoga Park, CA, USA), 100 units/ml penicillin, and 100 mg/ml streptomycin, and cells were non-treated (control) or treated with 10 ng/ml LPS from *Escherichia coli* (O111:B4, Sigma-Aldrich, Darmstadt, Germany), and cultured in poly-HEMA (Santa Cruz Biotechnology, Dallas, TX, USA)-coated plates for 4 days. After that, monocytes were purified by cell sorting using the same strategy as mentioned above. Alternatively, CD14+ monocytes were isolated using positive selection with CD14 magnetic Microbeads (Miltenyi Biotec, Bergisch Gladbach, Germany) and cultured in the same conditions as PBMCs. Purified monocytes were pelleted and stored until DNA or RNA extraction. Supernatants were collected and stored at − 80 °C until cytokine measurement.

The endotoxin tolerance status of the septic patients and healthy donors was evaluated by exposing whole blood to a stimulus of 5 ng/ml LPS, collecting supernatant after 3 h to determine the state of innate immune system (IIS) during initial infection.

### Cytokine measurements

The cytokine levels in whole blood were determined using the cytometric bead array (CBA) Flex Set (BD Biosciences, San Jose, CA, USA), following the manufacturer’s protocol. The collected data were analyzed with Flow Cytometric Analysis Program (FCAP) Array Software v3.0 (BD Biosciences). For in vitro experiments, the concentration of cytokines was measured from the cell culture supernatants using an enzyme-linked immunosorbent assay (ELISA), according to the manufacturer’s instructions (BioLegend, San Diego, CA, USA).

### DNA methylation profiling using universal bead arrays, bisulfite sequencing, and pyrosequencing

Infinium MethylationEPIC BeadChip (Illumina, Inc., San Diego, CA, USA) array were used to analyze DNA methylation. This platform allows > 850,000 methylation sites per sample to be interrogated at single-nucleotide resolution, covering 99% of reference sequence (RefSeq) genes. The samples were bisulfite-converted using EZ DNA Methylation-Gold™ Kit (Zymo Research, Irvine, CA, USA) and were hybridized in the array following the manufacturer’s instructions.

Each methylation data point was obtained from a combination of the Cy3 and Cy5 fluorescent intensities from the M (methylated) and U (unmethylated) alleles. For representation and further analysis, we used beta and *M* values. Beta value is the ratio of the methylated probe intensity to the overall intensity (the sum of the methylated and unmethylated probe intensities). The *M* value is calculated as the log2 ratio of the intensities of the methylated versus unmethylated probe. Beta values range from 0 to 1, in which 0 is no methylation and 1 is complete methylation, and were used to derive heatmaps and to compare DNA methylation percentages from bisulfite pyrosequencing experiments. For statistical purposes, the use of *M* values is more appropriate.

Bisulfite pyrosequencing was used to validate CpG methylation changes. DNA was isolated using ReliaPrep™ FFPE gDNA Miniprep System (Promega, Madison, WI, USA) for methylation array samples and with Maxwell® RSC Cultured Cells DNA Kit (Promega, Madison, WI, USA) for in vitro model. Bisulfite modification of genomic DNA isolated from monocytes was performed using EZ DNA Methylation-Gold™ Kit (Zymo Research, Irvine, CA, USA) following the manufacturer’s protocol. Primers for PCR amplification and sequencing were designed with the PyroMark® Assay Design 2.0 software (QIAGEN, Hilden, Germany). See list of primers in Additional file 2: Table S2. PCRs were performed with the IMMOLASE™ DNA polymerase PCR kit (Bioline Reagents Limited, London, UK), and the success of amplification was assessed by agarose gel electrophoresis. PCR products were pyrosequenced with the PyromarkTM Q24 system (QIAGEN, Hilden, Germany).

### Quality control, data normalization, and detection of differentially methylated and variable CpGs

Methylation array data were processed in the statistical language R using methods from the Bioconductor libraries minfi, lumi, and limma. Data quality was assessed using the standard pipeline from the minfi package. The data were Snoob-normalized and, after normalization, beta and *M* values were calculated. To exclude technical and biological biases, we developed a pipeline with several filters as removing CpGs with SNPs overlapped. To minimize the potential confounding influence of age and gender, we used these parameters as covariates.

In this study, we considered a probe to be differentially methylated if it had a methylation differential of 15% (Δ*β* ≥ 0.15) and when the statistical test was significant [*p* < 0.01 and false discovery rate (FDR) < 0.05]. In addition, we used the iEVORA algorithm [23] to designate a probe as differentially variable. This algorithm detects the homogeneity of variances using Bartlett’s test (FDR < 0.001) and then selects those probes whose *t* test is significant (*p* < 0.05 and FDR < 0.05) in order to regularize the variability test which is overly sensitive to single outliers.

Spearman’s correlation was used to correlate methylation changes with cytokine concentration. Spearman’s correlation coefficient is a nonparametric approach to measuring the strength of association of two variables being more reliable with non-linear data. We used the parameters specified in each section for Spearman’s analysis.

### Gene ontology analysis, transcription factor (TF) enrichment analysis, and chromatin state discovery and characterization (ChromHMM)

Gene ontology (GO) was analyzed using the Genomic Regions Enrichment of Annotations Tool (GREAT, version 3.0.0) (http://great.stanford.edu/public/html/). GREAT assigns biological meaning to a set of non-coding genomic regions by analyzing the annotations of the nearby genes [24]. For gene identification, we assigned a window that extends 5 kb upstream and 5 kb downstream from the differentially methylated CpG site. This window allows the analysis of CpGs located in regulatory regions distant to a TSS. Enrichment is showed as –log_10_ raw binomial *p* values.

We used the findMotifsGenome.pl program of the HOMER suite to look for motifs that are overrepresented in the target set relative to the background set (software v4.5). It was used to identify enrichment of TF binding motifs in the 500-bp window upstream and downstream of the differentially methylated CpG sites [25]. Annotated CpGs in the EPIC array were used as background.

Chromatin state discovery and characterization (ChromHMM algorithm) was used to analyze enrichment of the different chromatin states for the corresponding CpG sites [26]. The enrichment among chromatin states is defined using the 18-state ChromHMM model (Roadmap Epigenomics Integrative Analysis Hub, ChromHMM track of the UCSC Genome Browser) (http://www.roadmapepigenomics.org/) based on six chromatin marks (H3K4me3, H3K4me1, H3K27ac, H3K36me3, H3K27me3, and H3K9me3). A Fisher’s exact test was used to assign odds ratio and *p* value.

### Quantitative reverse transcription polymerase chain reaction (qRT-PCR)

RNA was isolated by Maxwell® RSC simplyRNA kit (Promega, Madison, WI, USA) and reverse-transcribed using the Transcriptor First Strand cDNA Synthesis Kit (Roche, Basel, Switzerland) according to the manufacturer’s instructions. qRT-PCR was performed in triplicate using LightCycler® 480 SYBR Green Mix (Roche, Basel, Switzerland). Expression values were normalized against the expression of the endogenous gene controls as *RPL38*. See list of primers in Additional file 2: Table S2.

### Statistical analysis

Data were analyzed with Prism version 6.0 (GraphPad). Statistical analyses were performed using the Mann-Whitney test, except as indicated. The levels of significance were as follows: *p < 0.05, **p < 0.01, ***p < 0.001.

## Results

### Monocytes from individuals who have undergone sepsis display an aberrant methylation signature

**Fig. 1:**
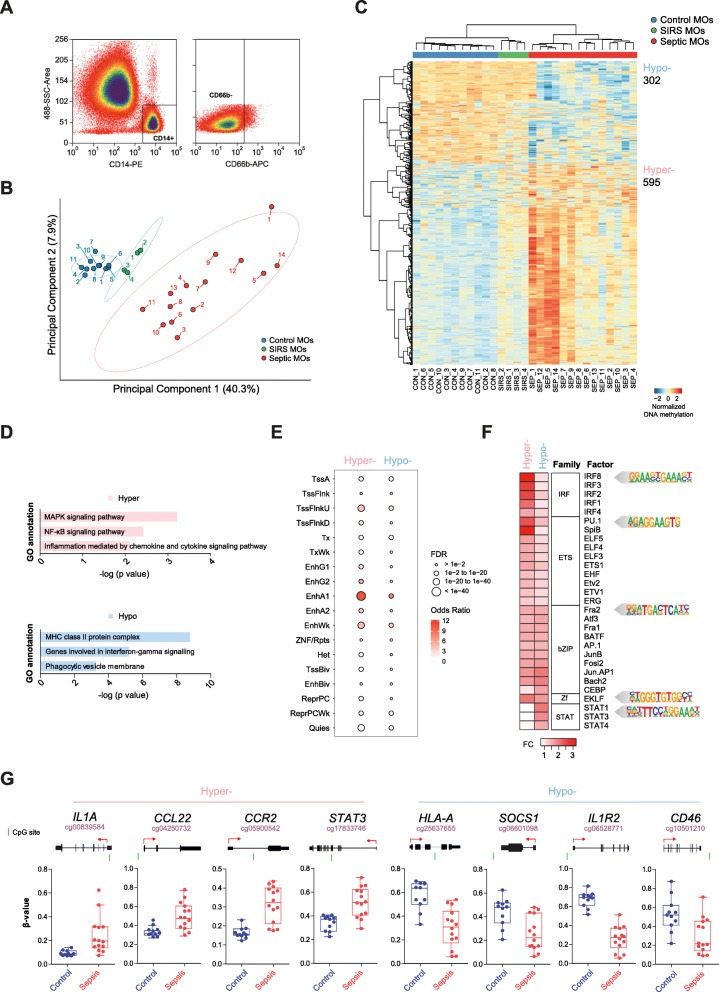
Global analysis of DNA methylation changes in septic monocytes. **a** Representative flow cytometry profiles indicating the sorting strategy and gates used in this study. Monocytes (MOs) (CD14+ CD66b−) were sorted from healthy controls and patients (SIRS and sepsis). **b** Principal component analysis (PCA) of methylation heatmap data for control, SIRS, and septic monocytes (in blue, green, and red respectively). **c** DNA methylation heatmap showing differentially methylated CpGs between controls (CON, blue) and patients with sepsis (SEP, red). The heatmap includes all CpG-containing probes displaying significant methylation changes (15% of differential of beta values, *p* < 0.01 and false discovery rate (FDR) < 0.05). A scale is shown at the bottom left ranging from − 2 (lower DNA methylation levels, blue) to + 2 (higher methylation levels, red). **d** Gene ontology (GO) analysis of genes associated with differentially methylated CpG sites showing the most relevant and significantly enriched categories resulting from the Genomic Regions Enrichment of Annotations Tool (GREAT). **e** Enrichment analysis of the different chromatin states for CpG sites corresponding to each methylation cluster (left to hypermethylation, right to hypomethylation). The relative enrichment of the different states is represented using the odds ratio. Dot size represents the FDR value. Tss, transcription start site; Enh, enhancer; Repr, repressed region; PC, Polycomb. **f** TF binding motif analysis of differentially methylated CpGs between control and sepsis. The panel shows fold change (FC), TF family and factor (selected TF with *p* ≤ 1e^−05^ for hypermethylated regions and *p* ≤ 1e^−03^ for hypomethylation). Motif logo is representative of the TF family. **g** Box plots showing *β*-values obtained from the DNA methylation array. We observed hypermethylation and hypomethylation in important immune system genes. The CpG sites are marked with a green line in the gene scheme placed on top of each graph, where the TSS is marked with a red arrow

We first performed DNA methylation screening on monocytes, sorted from peripheral blood as CD14+CD66b− cells (Fig. 1a and Additional file 3: Figure S1), from a cohort of 14 septic patients (Table 1 and Additional file 1: Table S1) and compared it with those sorted from a cohort of 11 healthy controls. We also included 4 patients with systemic inflammatory response syndrome (SIRS) following cardiac surgery. For the analysis, we used bead arrays to interrogate the DNA methylation status of > 850,000 CpG sites across the entire genome covering 99% of RefSeq genes. In the analysis, to minimize the potential confounding influence of age and gender (shown in Table 1), we used these parameters as covariates.

Principal component analysis (PCA) showed the two groups of monocytes from patients with sepsis and controls separated along the first principal component (Fig. 1b), with the SIRS in between. Overall, we observed a wider heterogeneity in septic monocytes than in control and SIRS monocytes (Fig. 1b), perhaps due to the diversity of infective bacteria (Additional file 1: Table S1). Monocytes from sepsis patients display 595 CpG sites with significantly higher methylation levels (hypermethylated) and 302 CpG sites with significantly lower methylation levels (hypomethylated) than control monocytes (Fig. 1c and Additional file 4: Table S3).

We performed gene ontology (GO) analysis to determine whether the differentially methylated genes were associated with relevant biological processes in sepsis. In the hypermethylated set, there was enrichment of important GO categories such as MAPK signaling pathway, NF-κB signaling pathway, and inflammation mediated by chemokine-cytokine signaling pathway. In the hypomethylated group, the functional categories were also relevant in the context of immune cell biology, including the MHC class II protein complex, genes involved in interferon-gamma (IFN-γ) signaling, and phagocytic vesicle membrane (Fig. 1d). The analysis of the chromatin states of differentially methylated CpG sites revealed the enrichment in active and weak enhancers (characterized by H3K4me1 and H3K27ac) for both the hyper- and hypomethylated sets and also for transcription start site (TSS) flanking regions in the case of the hypermethylated set (additionally marked by H3K4me3) (Fig. 1e).

We then inspected the enrichment of TF binding motifs among the two sets of differentially methylated CpG sites. We observed a significant overrepresentation of binding sites of the interferon regulatory factor (IRF) and ETS TF families in hypermethylated regions in septic monocytes (Fig. 1f). Previous reports have shown that ETS factors such as PU.1 can recruit DNA methyltransferases [27]. Hypermethylation could also antagonize the function of these TFs in endotoxin response [28]. Sequences around hypomethylated CpG sites were enriched for binding motifs of the signal transducer and activator of transcription (STAT) family (Fig. 1f). According to previous studies, the Janus kinase (JAK)/STAT pathway plays a critical role in protective immunity during sepsis via controlling cytokine responses (reviewed in [29]).

Inspection of individual genes among those containing differentially methylated CpG sites made it possible to identify some with essential functions in monocyte/macrophage biology and function. These included genes such as *IL1A*, *CCL22*, *CCR2*, and *STAT3* in the hypermethylated set and *HLA-A*, *SOCS1*, *IL1R2*, and *CD46* in the hypomethylated set (Fig. 1g and Table 2). IL1A is a pro-inflammatory cytokine. Different pro-inflammatory cytokines induce JAK activation, as well as the phosphorylation and activation of transcription activators STAT3, STAT5, and STAT6. CCL22 and CCR2 associate with leukocyte chemotaxis. HLA-A belongs to the MHC class I and is involved in antigen presentation. *SOCS1* encodes a member of the suppressor of cytokine signaling (SOCS) family that is involved in negative regulation of JAK/STAT cytokine signaling. Another example is *CD46*, encoding a costimulatory factor for T lymphocytes promoting T-regulatory 1 cells that suppress immune response through IL-10. All these examples highlight the relationship between the genes undergoing changes in DNA methylation and pathways related to the acquisition of endotoxin tolerance.

**Table 2:**
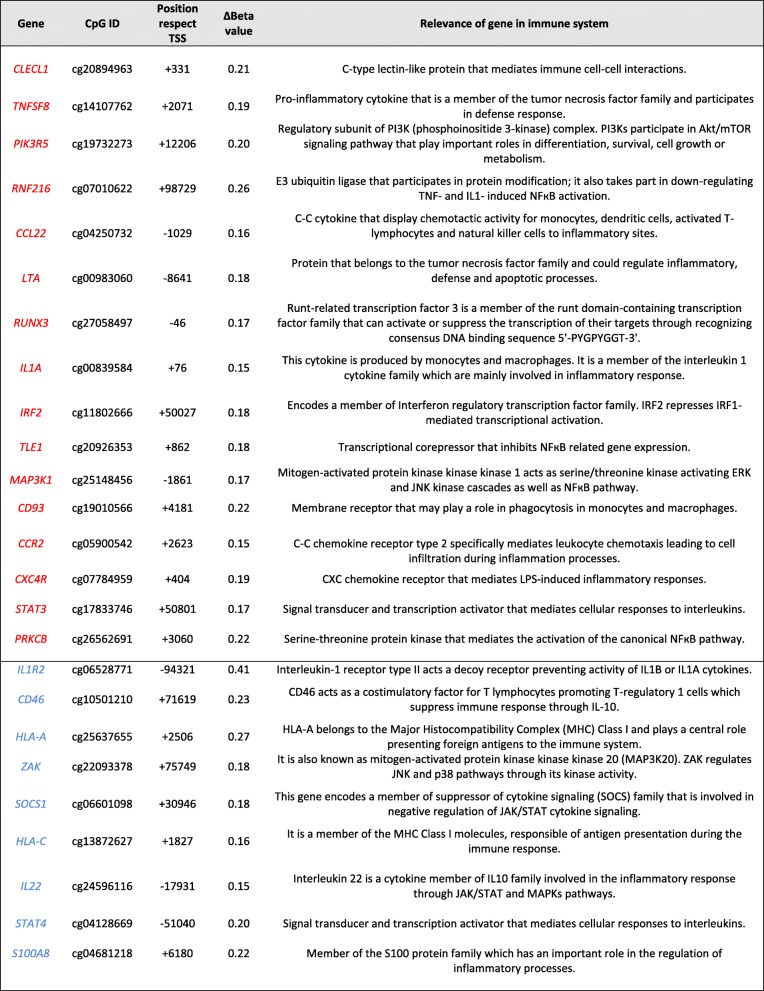
List of differentially methylated genes in septic monocytes

### Septic monocytes also display increased DNA methylation variability

**Fig. 2:**
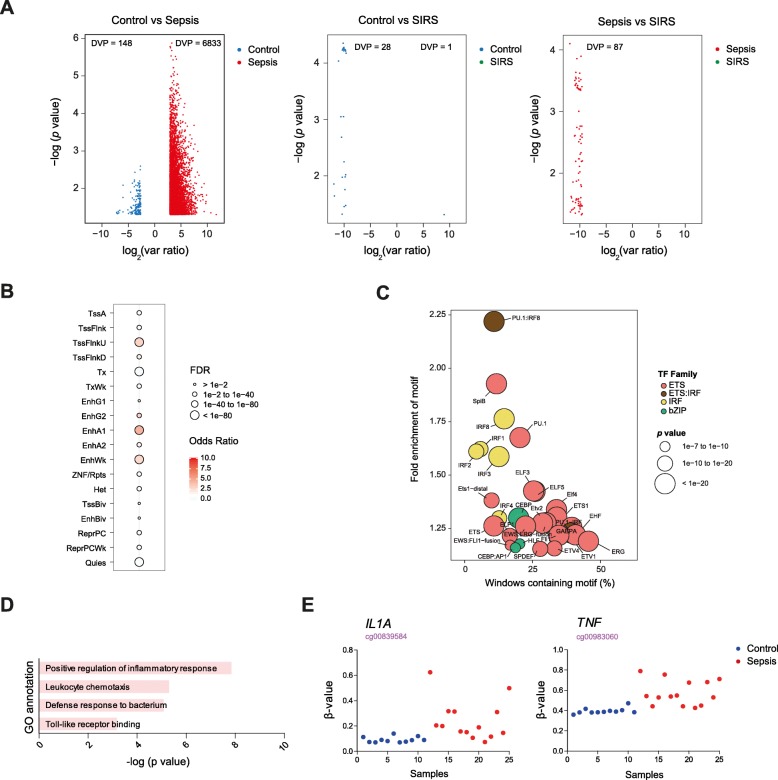
Analysis of differentially variable CpG positions (DVPs) in monocytes from patients with sepsis. **a** Volcano plot showing the *p* value vs the variance ratio for healthy control, SIRS, and sepsis-associated DVPs. DVPs were identified using the algorithm iEVORA. **b** Enrichment of sepsis-associated DVPs at 18 distinct chromatin states using ChromHMM. **c** TF binding motif analysis for sepsis-associated DVPs. Bubbles are colored according to TF family. The *p* value is indicated by bubble size (selected TF with *p* ≤ 1e^−07^ for DVP regions). **d** GO categories resulted from GREAT analysis for sepsis-associated DVPs of section (**a**). **e** Representative examples showing beta values of DVPs

As indicated above, our PCA analysis indicated a higher heterogeneity in the DNA methylation profiles of monocytes from patients with sepsis. We used a recently developed algorithm, named iEVORA [23], to determine significant differentially variable CpG positions (DVPs) at an FDR < 0.05 between monocytes from patients with sepsis and healthy individuals. By using this method, we not only could confirm the occurrence of a higher number of DVPs in monocytes from patients with sepsis (*n* = 6833) versus healthy controls (*n* = 148) (Fig. 2a) but also determine that many of these sites occur at genes that also display significant DNA methylation changes (Additional file 5: Table S4).

Similarly to our previous analysis of differentially methylated sites, sepsis-associated DVPs mainly occur at enhancers and also TSS flanking regions (Fig. 2b). Sequences surrounding these DVPs were highly enriched in binding motifs of ETS and IRF families (Fig. 2c), again highlighting the role of these TFs in the acquisition of the tolerogenic phenotype of septic monocytes.

GO analysis revealed an enrichment of sepsis-associated DVPs in positive regulation of inflammatory response, leukocyte chemotaxis, and defense response to bacterium and toll-like receptor binding (Fig. 2d). We found sepsis-associated DVPs in genes important for immune response against infection and hyperinflammation such as *IL1A* and *TNF* (Fig. 2e and Additional file 5: Table S4).

All this suggests that a variety of factors encompassing bacterial infection and sepsis are driving DNA methylation and phenotypic changes in these cells in a similar manner, regardless of the infecting bacteria or individual-specific clinical outcome of the individual.

### DNA methylation changes in monocytes from patients with sepsis correlate with increased IL-10 and IL-6 levels

Many of the characteristics of endotoxin-tolerized monocytes/macrophages resemble that of anti-inflammatory M2 macrophages [30, 31]. M2 macrophages show downregulated inflammatory cytokines (e.g., IL-12, TNFα) but upregulated anti-inflammatory cytokines (e.g., IL-10), scavenger receptor expression, and efficient phagocytosis. We wondered whether the DNA methylation patterns observed in monocytes from patients with sepsis might be associated with the generation of an immunosuppressive or tolerogenic environment in peripheral blood.

**Fig. 3:**
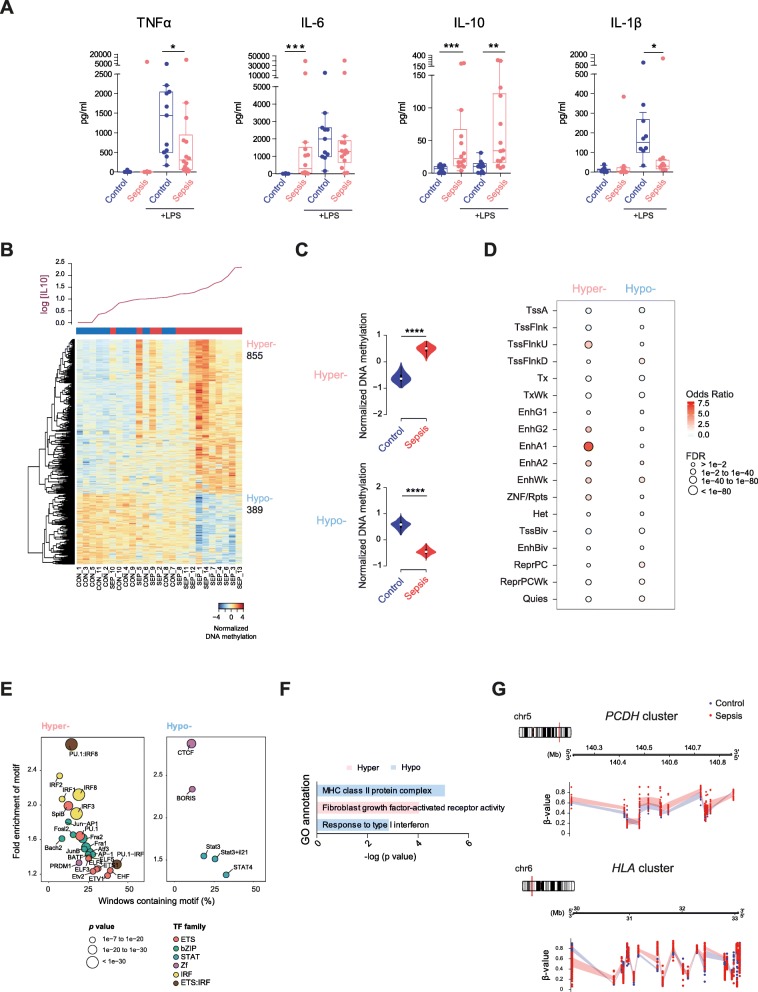
DNA methylation changes in septic monocytes parallel the increase of IL-10 levels. **a** Cytokine measurement using cytometric bead array (CBA) from control and septic PBMCs before and after LPS stimulation (*t* = 3 h). Box and whisker plots show median values. Mann-Whitney test was used to determine significance (*p < 0.05, **p < 0.01, and ***p < 0.001). **b** DNA methylation heatmap of CpG changes in relation to IL-10 basal concentration (represented on the top of the heatmap as log scale). Spearman’s correlation was used with *p* < 0.01, *r* > 0.5, and Δ*β* ≥ 0.15. A scale is shown at the bottom, wherein beta values range from − 4 (lower DNA methylation levels, blue) to + 4 (higher methylation levels, red). **c** Violin plots corresponding to the 5mC-normalized data for control and sepsis presented in the heatmap in the previous section. The median and the interquartile range are represented. **d** Chromatin state characterization of differentially methylated sites for section **b**. The relative enrichment of the different state assignments is representing using the odds ratio. FDR is represented by the size of the dots. **e** Bubble scatterplot of TF enrichment for hypermethylated and hypomethylated CpGs. The *x*-axis shows the percentage of windows containing the motif, and the *y*-axis shows the fold enrichment of the motif. Bubbles are colored according to TF family. *p* value is indicated by the bubble size (selected TF with *p* < 1e^−07^ for hypermethylated and hypomethylation regions). **f** GO categories resulted from GREAT analysis of differentially methylated CpGs related to IL-10 concentration. **g** Genomic tracks representing the clusters of differentially methylated CpGs for protocadherins (*PCDH*, upper scheme) and human leukocyte antigen (*HLA*, lower panel). Blue and red lines represent the confidence intervals for each average values. A window of ± 50,000 bp was used

To address this question, we first tested the levels of a panel of cytokines associated with acute sepsis in cultured PBMCs from patients with sepsis and compared it to those in control individuals. We identified significantly increased levels of IL-10 and IL-6 in patients with sepsis (Fig. 3a). We also examined the levels of cytokines following the exposure to LPS. This analysis showed that the majority of the septic patients had acquired tolerance, with decreased levels of pro-inflammatory TNFα, IL-1β, and IL-6, following exposure to LPS, and increased secreted levels of anti-inflammatory IL-10, indicating a higher degree of tolerogenic properties on septic monocytes (Fig. 3a), as previously reported [32].

Given the greater heterogeneity of the methylation profiles of septic samples with respect to those from healthy controls, we investigated whether this could also be related to the wider range of cytokine levels, such as those observed for IL-10 or IL-6. To this end, we first performed Spearman’s correlation between the DNA methylation data and the secreted IL-10 levels, for all patients with sepsis and control individuals. This analysis revealed that there are 855 CpG sites that become hypermethylated and 389 CpG sites hypomethylated in relation to increasing levels of IL-10 (*r* > 0.5; Δ*β* ≥ 0.15) (Fig. 3b and Additional file 6: Table S5). This analysis also showed that, for CpG sites associated with IL-10 levels, there is a significant difference of their median DNA methylation levels between septic and healthy controls (Fig. 3c), reinforcing the notion of the existence of differential methylation patterns between control and sepsis in addition to a contribution of IL-10 to the acquisition of such changes. Spearman’s correlation between the DNA methylation data and the IL-6 levels identified 2492 CpG sites becoming hypermethylated and 909 CpG sites hypomethylated in relation to increasing levels of IL-6 (*r* > 0.5; Δ*β* ≥ 0.1) (Additional file 7: Figure S2A and Additional file 8: Table S6).

CpG sites displaying changes in methylation in relation to IL-10 levels enriched for specific chromatin features (Fig. 3d). Specifically, we found enrichment for enhancers in both the hypermethylated and hypomethylated sets. We also investigated for this set of CpG sites the enrichment in TF binding motifs for these differentially methylated CpG sites. We observed that approximately 25% of the hypermethylated CpG sites have motifs for bZIP TF factor family (Jun-AP-1) and 25% of the hypomethylated CpG sites display motifs for STAT family members (Fig. 3e), suggesting that methylation might be driven by the stimulation of TLR by bacteria and the increased levels of IL-10, stimulating its receptor IL-10R. Interestingly, 15% of the hypomethylated sequences displayed enrichment of CTCF binding motifs, whose global occupancy has been linked to differential DNA methylation [33]. Similar results were obtained when we analyzed the data for the Spearman’s correlation with IL-6 levels (Additional file 7: Figure S2C and S2D).

GO analysis revealed distinct enriched biological processes for hypo- and hypermethylated CpG clusters in relation to IL-10 levels, including MHC class II protein complex and response to type I IFN, and fibroblast growth factor-activated receptor activity respectively (Fig. 3f). Of note, similar GO categories were also enriched for differentially methylated CpGs correlating to IL-6 levels (Additional file 7: Figure S2B).

Our data show that many members of Wnt signaling pathway (*WNT3A*, *WNT6*, and *AXIN2* among others) (Additional file 6: Table S5) display a gain of methylation in septic monocytes compared with their healthy counterparts, highlighting the potential link between aberrant DNA methylation and the Wnt pathway. In fact, cumulative evidence supports the role of the Wnt pathway in the regulation of the macrophage-mediated inflammatory response in sepsis [34], in which Wnt3a and Wnt6 reduce TNFα secretion and promote the differentiation towards an M2 anti-inflammatory phenotype attenuating the immune response [35]. Furthermore, we found a large genomic region that also displayed DNA hypermethylation in which it predominantly covered CpG sites in the three tandem gene clusters of protocadherin (*PCDHA*, *PCDHB*, and *PCDHG*) (Fig. 3g) (Additional file 6: Table S5). This region has previously been reported to undergo aberrant DNA hypermethylation in cancer and in other disorders [36, 37]. Moreover, recent studies have identified mechanisms by which PCDHs can regulate the Wnt pathway (reviewed in [38]), which further corroborates the Wnt pathway as a putative therapeutic target for the patient treatment.

Regarding the hypomethylated CpG sites, we found an enrichment in genes involved in the IFN-γ pathway, which is essential for antimicrobial defense and restoring monocyte deactivation in patients with sepsis [39]. Remarkably, among the CpG sites displaying changes in methylation in relation to IL-10 levels, we identified 23 CpG sites within the HLA cluster, which is also induced by the IFN-γ and JAK/STAT pathway [40] (Fig. 3g).

### Monocytes exposed to LPS undergo DNA methylation changes in parallel with the acquisition of endotoxin tolerance

**Fig. 4:**
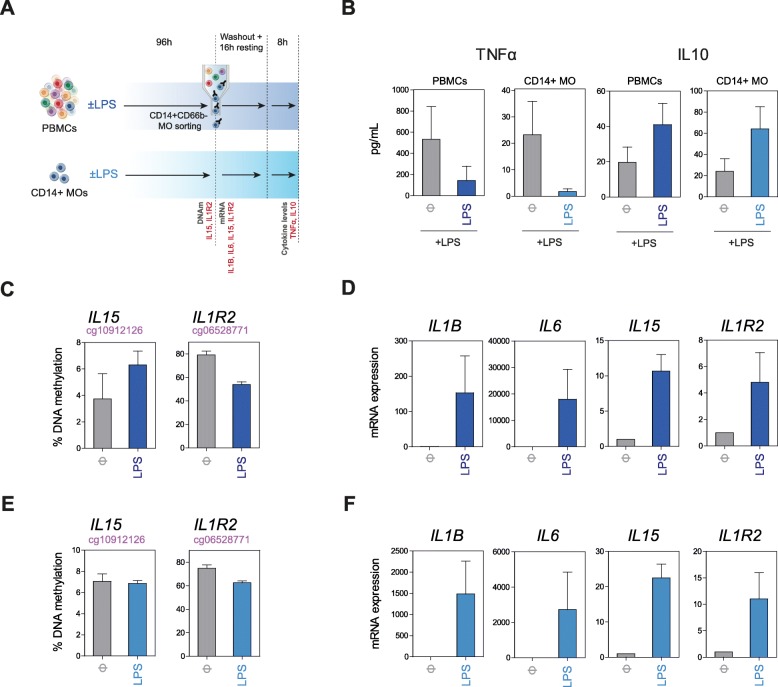
In vitro exposure to LPS induces the acquisition of tolerance and DNA methylation changes similar to those observed in sepsis. **a** Schematic diagram depicting our in vitro model for sepsis. PBMCs were cultured with or without LPS during 4 days, and then, monocytes were sorted as CD14+CD66b− cells for subsequent analyses. In parallel, monocytes were isolated with magnetic CD14 antibody and cultured in the same conditions. **b** TNFα and IL-10 production, as determined by ELISA, from PBMCs or monocyte supernatants, following washout and resting following the 4 initial days (with/without LPS) and an 8-h exposure to LPS, as indicated in the scheme in **a**. **c** Bisulfite pyrosequencing of selected hypermethylated (*IL15*) and hypomethylated (*IL1R2*) genes in sorted CD14+CD66b− monocytes from PBMCs in the in vitro sepsis model. **d** *IL1B*, *IL6*, *IL15*, and *IL1R2* mRNA levels were analyzed by quantitative RT-PCR using *RPL38* as control in the same sorted monocytes. **e** Bisulfite pyrosequencing of *IL15* and *IL1R2* in CD14+ monocytes in the in vitro sepsis model. **f** *IL1B*, *IL6*, *IL15*, and *IL1R2* mRNA levels analyzed by quantitative RT-PCR in CD14+ monocytes

Our results suggest that TLR stimulation and the inflammatory environment generated in the context of systemic bacterial infection are able to induce DNA methylation changes in monocytes. First, the specific DNA methylation profiles of monocytes from patients with sepsis associate with IL-10 and IL-6 levels. Second, there is an enrichment of binding motifs for AP-1 and STATs. This suggests that both the stimulation of TLRs, the resulting inflammatory conditions, and subsequent anti-inflammatory signals, participate in shaping the generation of aberrant methylation profiles which might modulate and stabilize the phenotype of monocytes following a septic episode. We therefore explored the possibility of inducing in vitro DNA methylation changes observed in such conditions by exposing in parallel PBMCs and monocytes from healthy individuals to LPS, and compare it with PBMCs and monocytes without such stimulation (Fig. 4a). We cultured the cells for 4 days and measured the acquisition of tolerance. In the case of PBMCs, we isolated monocytes, sorted as CD14+CD66b− cells after these 4 days (Fig. 4a, top). These experiments were performed in the presence of poly-2-hydroxyethyl methacrylate (poly-HEMA), an agent that restricts the attachment of the monocytes to the plates and therefore their differentiation to macrophages, to prevent the occurrence of vast changes in DNA methylation [19, 20]. Enzyme-linked immunosorbent assays (ELISA) revealed in both cases the acquisition of tolerance following the initial encounter with LPS. Specifically, these assays showed decreased levels of TNFα and increased levels of IL-10 in a second LPS exposure for those cells that had a first exposure to LPS (Fig. 4b). We then performed pyrosequencing of a selection of hypo- and hypermethylated genes in septic monocytes. Our analysis revealed that the in vitro stimulation with LPS is able to induce DNA methylation changes in these genes (Fig. 4c) with a similar trend to the changes observed in patients with sepsis when PBMCs were exposed to LPS (followed by monocyte purification). In the case of monocytes directly exposed to LPS, we were able to stimulate only demethylation of the *IL1R2* gene, suggesting that perhaps signals from other cell types are necessary to induce gains of methylation following LPS treatment. Interestingly, changes in DNA methylation at the aforementioned sites occurred in association with changes in gene expression of these genes (Fig. 4d, e) in the two sets of samples exposed to LPS, and not in controls. In addition, we also observed an increase in mRNA levels of pro-inflammatory cytokines (IL-1β and IL6) in LPS-treated cells. These results proved that both gains and losses of DNA methylation and expression in septic monocytes are the result of TLR stimulation and the generation of an inflammatory environment and are associated with the acquisition of a tolerized state in monocytes.

### Organ dysfunction associates with DNA methylation changes

**Fig. 5:**
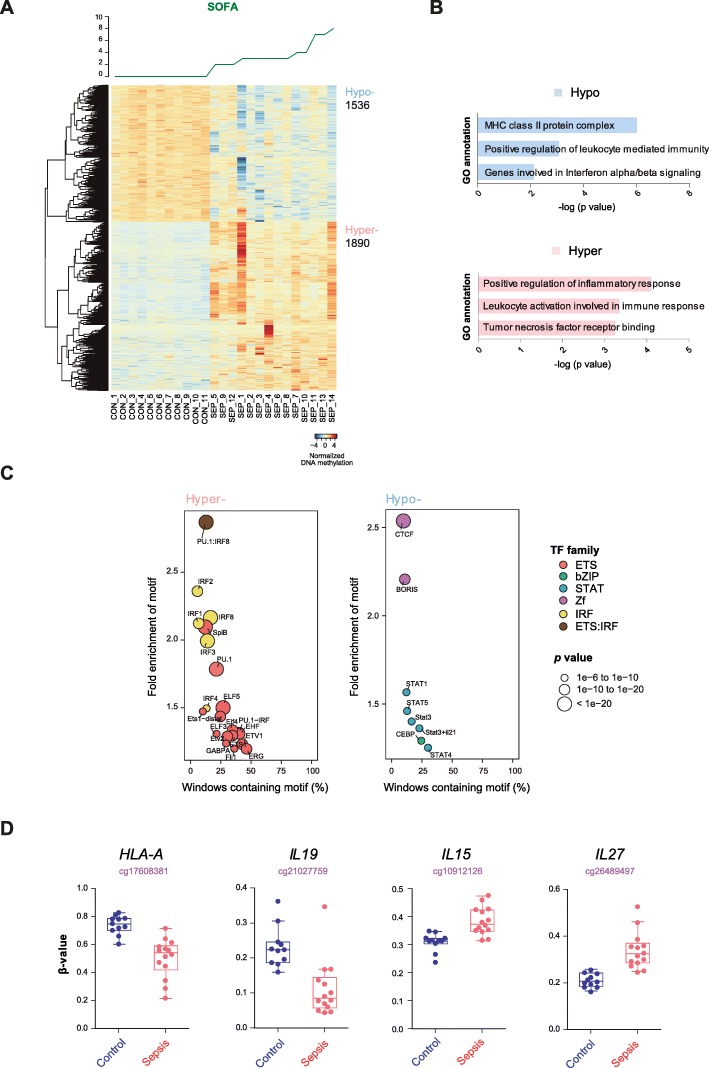
DNA methylation changes in septic monocytes parallel organic damage. **a** DNA methylation heatmap of CpG changes in relation to the SOFA score (represented on the top of the heatmap). Spearman’s correlation was used with *p* < 0.01, *r* > 0.6. A scale is shown at the bottom, wherein beta values range from − 4 (lower DNA methylation levels, blue) to + 4 (higher methylation levels, red). **b** GO categories resulted from GREAT analysis of differentially methylated CpGs related to SOFA. **c** Bubble scatterplot of TF enrichment for hypermethylated and hypomethylated CpGs. The *x*-axis shows the percentage of windows containing the motif, and the *y*-axis shows the fold enrichment of the motif. Bubbles are colored according to TF family. *p* value is indicated by bubble size (selected TF with *p* ≤ 1e^−06^ for hypermethylated and hypomethylation regions). **d** Box plots showing *β*-values obtained of genes significantly correlating with the SOFA score

We finally tested DNA methylation profiles in relation to SOFA, the main score used to assess organ dysfunction. When using Spearman correlation, we determined that there are 1890 CpG sites that become hypermethylated and 1536 CpG sites hypomethylated in relation to increasing SOFA (*p* value < 0.01; *r* > 0.6) (Fig. 5a and Additional file 9: Table S7). GO analysis revealed that DNA methylation changes in relation to SOFA affect inflammatory response and antigen presentation in a similar manner than previous comparisons (Fig. 5b). We also investigated for this set of CpG sites the enrichment in TF binding motifs for these differentially methylated CpG sites. We obtained similar sets of TF binding motifs (Fig. 5c) like the ones observed for the correlations with IL-10 and IL-6, suggesting the participation of these inflammatory cytokines in the acquisition of DNA methylation changes in relation to organic damage. Finally, individual inspection of the lists revealed the association of several relevant molecules including HLA-A, IL19, IL15, and IL27 (Fig. 5d). Altogether, this analysis suggested that the DNA methylation changes associated with organic damage involve similar changes to those observed in relation to inflammatory cytokines.

## Discussion

Our study demonstrates for the first time the existence of DNA methylation alterations in human monocytes from individuals following a sepsis episode in relation to the acquisition of a tolerized phenotype, paralleling data obtained in a mouse model [41]. Most notably, changes occur in genes relevant to the function of these cells including the interferon-gamma-mediated pathway and MHC class II proteins. On the one hand, the observed methylation changes in patients with sepsis suggest their participation among the mechanisms leading to the generation of an aberrant phenotype of these cells. On the other hand, correlation analyses of the DNA methylation profiles in relation to IL-10 and IL-6 levels, which are increased in patients with sepsis, suggest a potential mechanism downstream to these cytokines participating in the defective generation of DNA methylation alterations. Furthermore, in vitro analysis of the influence of bacterial LPS and inflammatory context in determining the acquisition of DNA methylation alterations in monocytes also shows how these changes associate with aberrant transcriptional levels of dysregulated genes. Finally, our analysis shows increasing changes in DNA methylation in relation to organ dysfunction.

Epigenetic factors play a role in the acquisition of endotoxin tolerance. In fact, seminal studies by the teams of Netea, Logie, and Stunnenberg have shown that the transcriptional inactivity in response to a second LPS exposure in tolerized macrophages is accompanied by a failure to deposit active histone marks at promoters of tolerized genes [17]. It has also been reported that leukocytes of patients with sepsis have defects in important metabolism pathways and, interestingly, these immunometabolic defects were partially restored by therapy with recombinant IFN-γ [7].

The aforementioned studies paid less attention to DNA methylation changes, as they appeared to be less prevalent than those at occurring in histone modifications. However, DNA methylation is a relatively more stable epigenetic mark than histone modifications. That property makes this modification worth of study: firstly, because it might have a long-term contribution to the tolerized state of monocytes, and secondly, because it could potentially be used as a marker, if associations with patient prognosis and/or progression were found.

**Fig. 6:**
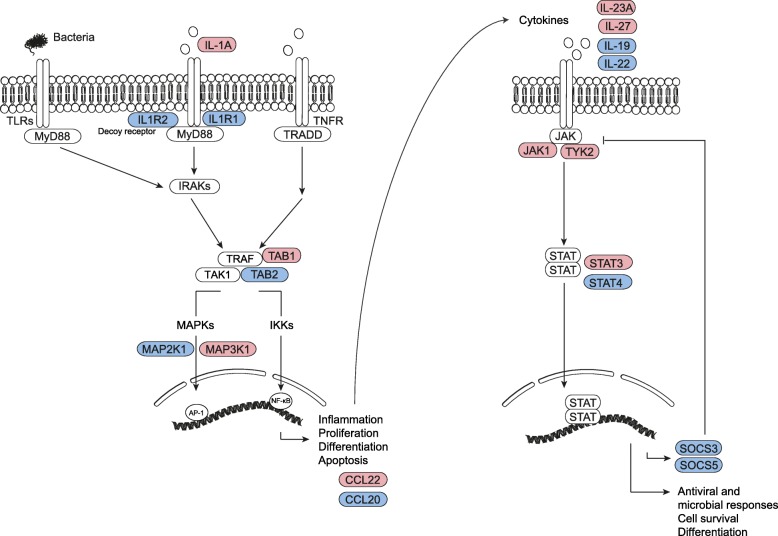
Scheme depicting important signaling pathways related to immunity and sepsis. Molecules whose encoding genes displayed DNA methylation alterations in this study are shown in red and blue for hypermethylation and hypomethylation respectively. The following proteins/genes are represented in the figure: TLR, Toll-like receptor; MyD88, myeloid differentiation primary response 88; IRAK, interleukin-1 receptor-associated kinase; IL1R, interleukin-1 receptor; IL-1A, interleukin 1 alpha; TNFR, tumor necrosis factor receptor; TRADD, TNFR1-associated death domain; TRAF, TNF receptor-associated factor; TAK1, transforming growth factor (TGF) beta-activated kinase 1; TAB2, TGF beta-activated kinase 1 binding protein 2; MAPK, mitogen-activated protein kinase; IKK, IκB kinase; AP-1, activator protein 1; NF-кB, nuclear factor kappa B; CCL20, C-C motif chemokine ligand 20; CCL22, C-C motif chemokine ligand 22; IL-23A, interleukin 23A; IL-19, interleukin 19; IL-27, interleukin 27; IL-22, interleukin 22; JAK1, Janus kinase 1; STAT, signal transducer and activator of transcription; TYK2, tyrosine kinase 2; SOCS, suppressor of cytokine signaling. In this scheme, we have selected CpG sites/genes with a minimum 10% of differential of beta values, *p* < 0.01 and false discovery rate (FDR) < 0.05

Many of the genes displaying differentially methylated CpG sites between patients with sepsis and controls occur within a limited number of pathways relevant to the stimulation associated with bacterial infection (Fig. 6). For instance, genes experiencing changes in methylation such as *IL1A*, *IL1R2*, *IL1R1*, *TAB2*, *TAB1*, *MAP 2K1*, and *MAP 3K1* occur within the same signaling pathway, which is also merging from the signals downstream to TNF. On the other hand, several of the genes encoding for cytokines like *IL27*, *IL23A*, *IL19*, and *IL22* also display CpG sites undergoing methylation changes, as well as genes encoding elements downstream to it, such as *TYK2*, *JAK1*, *STAT*3 and 4, and *SOCS3* and *SOCS5*. The relationship between all these genes suggests two possibilities: that DNA methylation changes at these sites have a causal effect in determining the activation or repression of the associated genes or, alternatively, that the monocyte methylome is acting as a sensor of the activation of these pathways through additional or alternative mechanisms.

In this respect, our results show that monocytes from patients with sepsis harbor an aberrant DNA methylation signature that is related with the abnormal environment derived from such inflammatory condition. Furthermore, we have determined that DNA methylation changes correlate with increased IL-10 and IL-6 levels and that those changes are functionally annotated to genes belonging to the Wnt and IFN-γ signaling pathways. In this respect, it has been demonstrated, as mentioned above, that IFN-γ-based therapy can partially restore the defective metabolic changes occurring in leukocytes from patients with sepsis [7].

Finally, our investigation demonstrates the sensitivity of monocytes to translate environmental changes into more stable changes at the transcriptional level through DNA methylation. Given that sepsis is associated with the generation of a particular cytokine environment [42], our results reinforce the notion that epigenetic changes are related to the maintenance of the dysregulated immune response following an episode of sepsis. However, with the existing data generated in this study, we cannot distinguish whether these epigenetic changes are a cause or a consequence: in other words, whether the DNA methylation changes are caused by these individuals’ infection history, perhaps influenced by the environment, or whether environmental factors cause the generation of the aberrant DNA methylation signature, which is then accompanied by immune responses that are secondary to the sepsis. It is likely that the aberrant DNA methylation (and expression signature) generated as a result of the particular cytokine milieu generated under the sepsis episode contributes to perpetuating the tolerized state of these monocytes. Whether those changes also affect bone marrow monocyte progenitors giving long-lasting reprogramming, as occurs with trained immunity [43], remains to be investigated. Interestingly, the observed changes appear to be reflective of the infection, as it suggests the data obtained for SIRS patients (even if it is a small cohort), which are also characterized by inflammation and organ dysfunction.

A potential limitation of our study is the size and characteristics of the cohort. In future studies, it would be necessary to use vaster cohorts, including patients with a representative number of Gram-positive and Gram-negative bacteria and patients at different stages following the sepsis episode. However, the size and features of our cohort, on the other hand, indicate that common changes in DNA methylation are associated with sepsis regardless of the infective bacteria. The identification of specific DNA methylation markers associated with the infecting organism of the clinical outcome of the patient will surely be useful for predicting the evolution of the patients and perhaps their clinical management.

## Conclusions

In the present study, we have shown that patients with sepsis undergo widespread changes in the methylome of their circulating monocytes in parallel with the acquisition of endotoxin tolerance. Thousands of changes are associated with the aberrant levels of IL-10 and IL-6, as well as with organ dysfunction. Stimulation of the Toll-like receptor in monocytes induces similar changes in DNA methylation and expression, concomitant with the acquired tolerance that points to a major role in the stabilization of a tolerized phenotype through these alterations. Our results open up possibilities not only to use DNA methylation as a marker for disease but also for understanding its role in the acquisition of the aberrant phenotype of these cells.

## Supplementary information

**Additional file 1: Table S1.** Clinical features of septic patients, SIRS and healthy controls included in this study.

**Additional file 2: Table S2.** Primers sequences.

**Additional file 3: Figure S1.** Purification and quality of monocytes. (a) Flow cytometry profiles indicating the sorting strategy and gates used in the study. (b) Cell type deconvolution of the hybridized samples using Houseman algorithm.

**Additional file 4: Table S3.** List of hypermethylated and hypomethylated genes in septic monocytes (р-value< 0.01; FDR < 0.05; ∆β ≥ 0.15).

**Additional file 5: Table S4.** List of differentially variable CpG positions (DVPs).

**Additional file 6: Table S5.** List of hypermethylated and hypomethylated CpGs related to IL-10 cytokine.

**Additional file 7: Figure S2.** DNA methylation changes in septic monocytes parallel the increase of IL-6 levels. (a) DNA methylation heatmap of CpGs changes in relation to IL-6 basal concentration. Spearman’s correlation was used with *p* < 0.01, r > 0.5 and differential β-value ≥0.1. A scale is shown at the bottom, wherein beta values range from − 4 (lower DNA methylation levels, blue) to + 4 (higher methylation levels, red). (b) GO categories for differentially methylated CpGs of section (a). (c) Enrichment of differentially hyper- and hypo-methylated CpGs among chromatin states, defined using the 18-state ChromHMM model. (d) HOMER motif analysis for methylation changes. The x-axis shows the percentage of windows containing the motif and the y-axis shows the fold enrichment of the motif. Bubbles are colored according to TF family. *p* value is indicated by bubble size (TF with *p* < 1e^−15^ for hypermethylated regions and *p* ≤ 1e^−5^ for hypomethylation were represented).

**Additional file 8: Table S6.** List of hypermethylated and hypomethylated CpGs related to IL-6 cytokine.

**Additional file 9: Table S7.** List of hypermethylated and hypomethylated CpGs related to SOFA.

## Data Availability

Methylation array data for this publication have been deposited in NCBI’s Gene Expression Omnibus. Lorente-Sorolla C, García-Gómez A, Català-Moll F, Toledano V, Ciudad L, Avendaño-Ortiz J, Maroun-Eid C, Martín-Quirós A, Martínez-Gallo M, Ruiz-Sanmartín A, García del Campo Á, Ferrer-Roca R, Ruiz-Rodriguez JC, Álvarez-Errico D, López-Collazo E, Ballestar E. Inflammatory cytokines and organ dysfunction associate with the aberrant DNA methylome of monocytes in sepsis. Methylation profiling by array of CD14+CD66bneg monocytes of 11 healthy donors, 4 SIRS, and 14 septic patients datasets. GEO Series accession number GSE138074.

## Abbreviations

AP-1: Activator protein-1
CBA: Cytometric bead array
CTF/NF1: CCAAT box-binding transcription factor/nuclear factor I
DNMT: DNA (cytosine-5)-methyltransferase
DVPs: Differentially variable CpG positions
ELISA: Enzyme-linked immunosorbent assay
FDR: False discovery rate
GO: Gene ontology
HDAC: Histone deacetylase
HLA: Human leukocyte antigen
IKK: IκB kinase
IL: Interleukin
IFN-γ: Interferon-gamma
IRF: Interferon regulatory factor
JAK: Janus kinase
LPS: Lipopolysaccharide
MAPK: Mitogen-activated protein kinase
MHC: Major histocompatibility complex
MyD88: Myeloid differentiation primary response 88
NF-кB: Nuclear factor kappa B
PBMCs: Peripheral blood mononuclear cells
PCA: Principal component analysis
PCDH: Protocadherins
poly-HEMA: Poly-2-hydroxyethyl methacrylate
SNP: Single-nucleotide polymorphism
SOCS: Suppressor of cytokine signaling
STAT: Signal transducer and activator of transcription
TET2: Ten-eleven translocation methylcytosine dioxygenase 2
TF: Transcription factor
TGF: Transforming growth factor
TLR: Toll-like receptor
TNF: Tumor necrosis factor
TRAF: TNF receptor-associated factor
TSS: Transcription start site
TYK2: Tyrosine kinase 2
