# Supplementary figures and images for: Inflammatory cytokines and organ dysfunction associate with the aberrant DNA methylome of monocytes in sepsis
Source: Genome Med. 2019 Oct 29;11:66. doi: 10.1186/s13073-019-0674-2 (PMC6820973; doi:10.1186/s13073-019-0674-2)

Figure S1

A

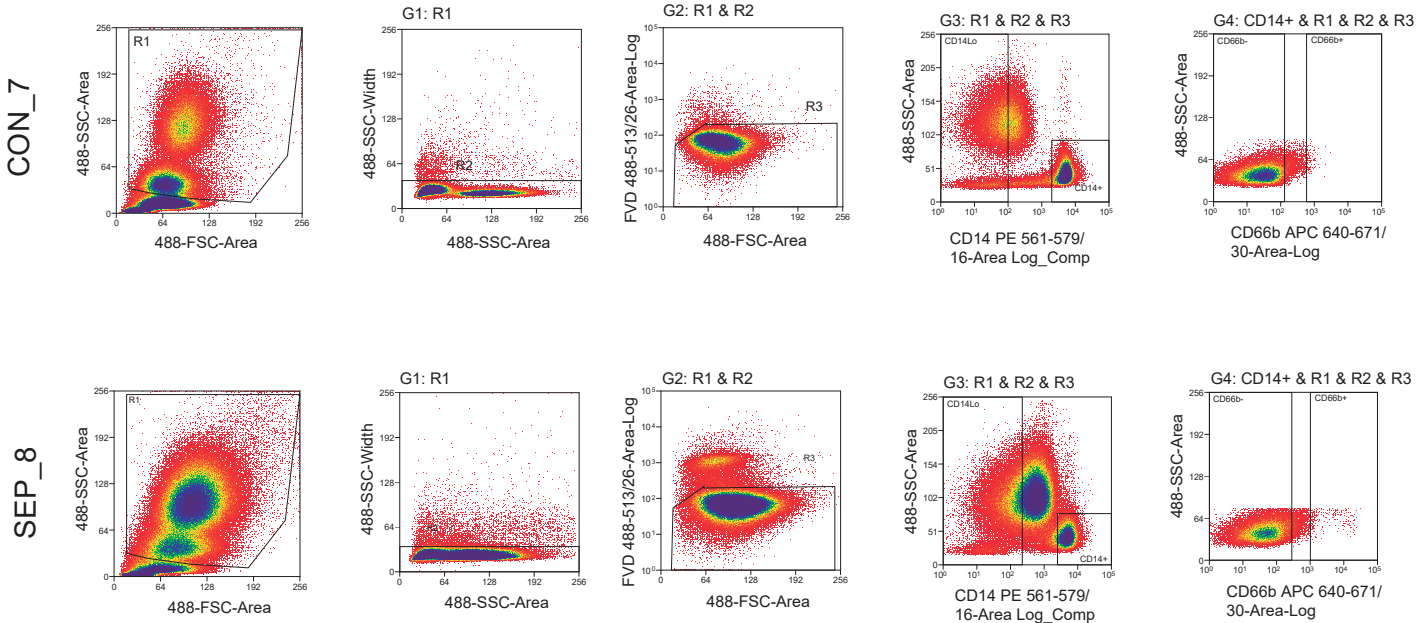

B

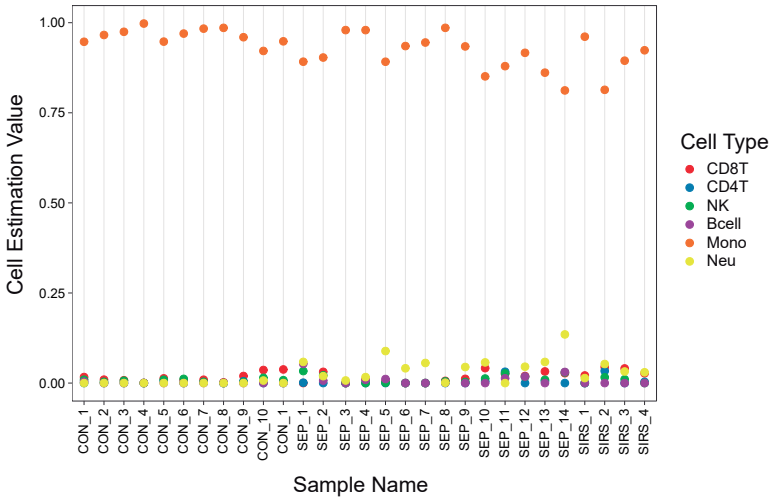

Supplement: Supplementary file 3 — Additional file 3: Figure S1. Purification and quality of monocytes. (a) Flow cytometry profiles indicating the sorting strategy and gates used in the study. (b) Cell type deconvolution of the hybridized samples using Houseman algorithm. [file 13073_2019_674_MOESM3_ESM.pdf]

Figure S2

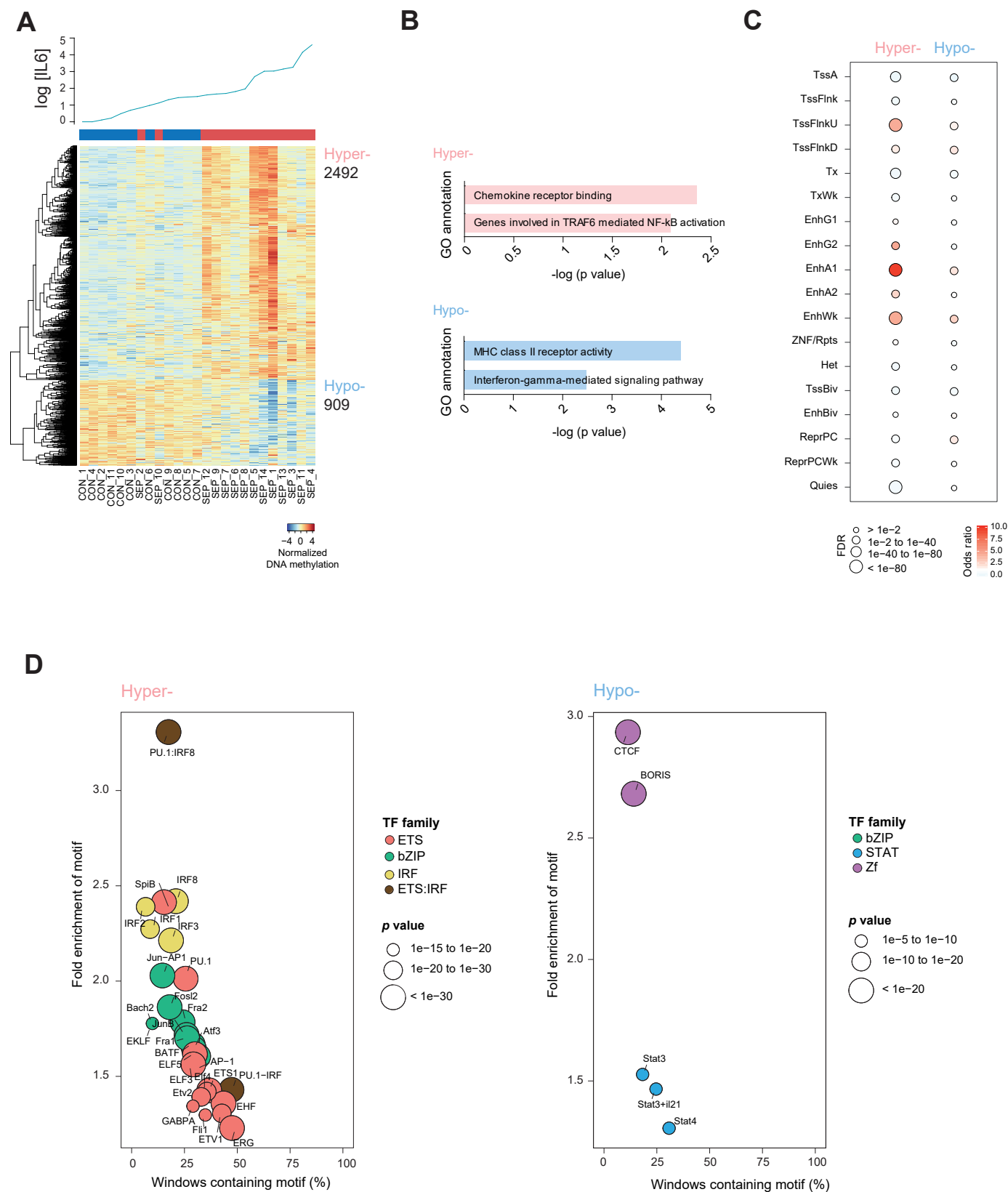

Supplement: Supplementary file 7 — Additional file 7: Figure S2. DNA methylation changes in septic monocytes parallel the increase of IL-6 levels. (a) DNA methylation heatmap of CpGs changes in relation to IL-6 basal concentration. Spearman’s correlation was used with p < 0.01, r > 0.5 and differential β-value ≥0.1. A scale is shown at the bottom, wherein beta values range from − 4 (lower DNA methylation levels, blue) to + 4 (higher methylation levels, red). (b) GO categories for differentially methylated CpGs of section (a). (c) Enrichment of differentially hyper- and hypo-methylated CpGs among chromatin states, defined using the 18-state ChromHMM model. (d) HOMER motif analysis for methylation changes. The x-axis shows the percentage of windows containing the motif and the y-axis shows the fold enrichment of the motif. Bubbles are colored according to TF family. p value is indicated by bubble size (TF with p < 1e−15 for hypermethylated regions and p ≤ 1e−5 for hypomethylation were represented). [file 13073_2019_674_MOESM7_ESM.pdf]
